# Successful Implantation of a Coronary Stent Graft in a Peripheral Vessel

**DOI:** 10.1155/2015/725168

**Published:** 2015-08-25

**Authors:** Alexander Hess, Britta Vogel, Benedikt Kohler, Oliver J. Müller, Hugo A. Katus, Grigorios Korosoglou

**Affiliations:** Department of Cardiology, Angiology and Pneumology, University of Heidelberg, Im Neuenheimer Feld 410, 69120 Heidelberg, Germany

## Abstract

Peripheral artery disease (PAD) is a complex, often underdiagnosed illness with rising prevalence in western world countries. During the past decade there has been a rapid advance especially in the field of endovascular treatment of PAD. Here we present for the first time a case reporting on the placement of coronary stent graft in a peripheral vessel for the management of a peripheral side branch perforation. Interventional angiologists or radiologists may consider such an option for complication management after injury of smaller vessels during peripheral percutaneous interventions. Further specialization and novel options of complication management as described in our case may shift the treatment from surgical to even more endovascular treatment procedures in the future.

## 1. Introduction

Peripheral artery disease (PAD) is a complex, often underdiagnosed illness with rising prevalence in western world countries [1]. Patients suffering from PAD present with a broad spectrum of symptoms ranging from asymptomatic vascular disease over intermittent claudication to critical limb ischemia (CLI). The overall life expectancy of patients with symptomatic PAD is 80% during 5 years of follow-up [2, 3]. CLI has a significant worse prognosis with an amputation rate of 14–20% and a death rate of 25% within the first year after diagnosis and 50% within five years [4].

Treatment of PAD involves life style modification (e.g., smoking cessation and physical exercise), consequent risk factor control (e.g., statin use), antithrombotic treatment, and endovascular or surgical revascularization. During the past decade there has been a rapid advance especially in the field of endovascular treatment of PAD, contributing to significant reduction of symptoms and improvement of outcomes in such patients. Thus, the number of major amputations decreases, with increasing rates of successful endovascular procedures within the last decade [5]. The recent ERASE study [6], on the other hand, showed that combining endovascular revascularization with supervised exercise training resulted in substantial improvement of clinical symptoms in patients with intermittent claudication. Despite all these technical advances with endovascular treatment option, complications during such procedures may still occur and their appropriate management remains a challenge for clinicians. One of the most feared complications is bleeding, which can lead to large painful hematoma or even to compartment syndrome.

## 2. Case Presentation

**Figure 1:**
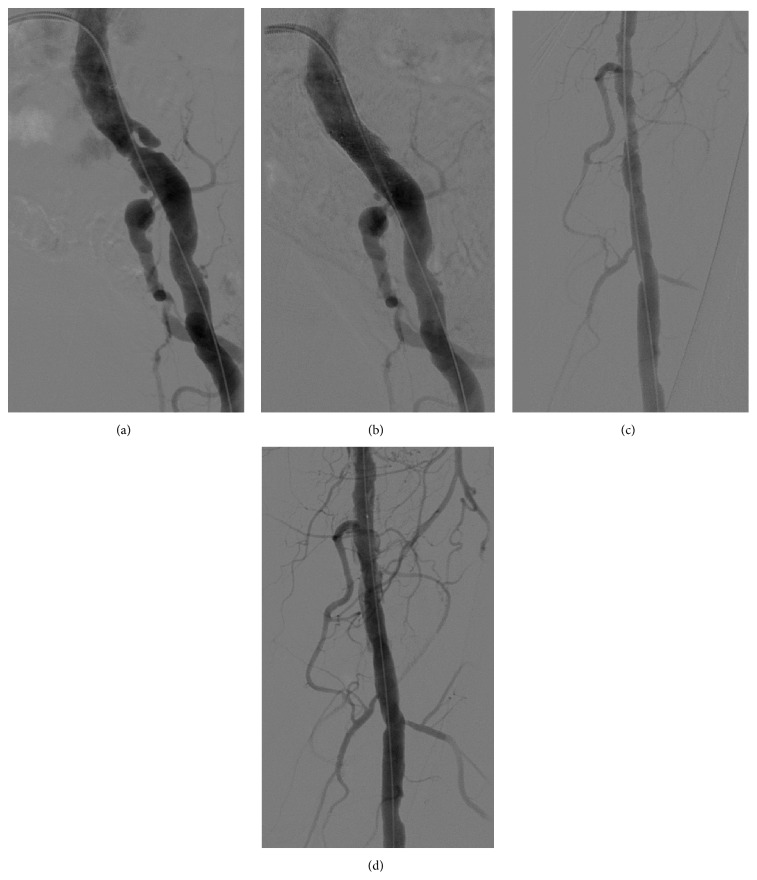
Left common iliac artery before (a) and after (b) percutaneous transluminal angioplasty and placement of one bare metal stent. Left superficial femoral artery before (c) and after (d) percutaneous transluminal angioplasty with a drug eluting balloon.

**Figure 2:**
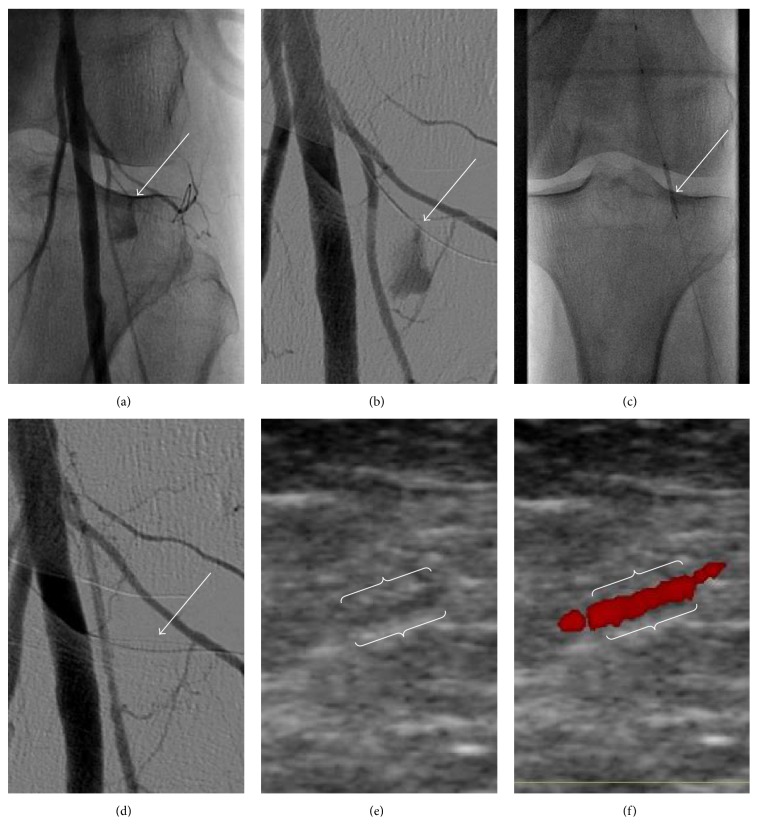
Perforation of a small side branch of the popliteal artery presumably by the 0.035′′ Terumo guide wire (white arrow in (a)). A 0.014′′ wire was subsequently inserted into this side branch (b), and bleeding was stopped after placement of a coronary Direct-Stent stent graft (c and d). Using colour doppler ultrasound the stent graft (white brackets) could be visualized one day after implantation, exhibiting normal blood flow (e and f).

An 88-year-old patient suffering from Fontaine stage IIb peripheral artery disease of his left leg was referred for interventional treatment in our angiology department. Using digital subtraction angiography (DSA) high grade lesions were identified in both his left common iliac and left superficial femoral artery, which were treated by percutaneous transluminal angioplasty (PTA) and placement of a bare metal (12*∗*40 mm Dynamic, Biotronik, Berlin, Germany) stent and by drug-eluting PTA (6.0*∗*120 mm, INPACT Admiral,* Medtronic*, Minneapolis, USA), respectively, using a 0.035′′ Terumo Stiff hydrophilic guide wire (Figures 1(a)–1(d)). A minor not flow-limiting dissection was treated with DEB to prevent restenosis. At the end of the procedure a cine angiography of the leg and of the popliteal artery was performed, which revealed that presumably during the intervention a very small side branch of the popliteal artery was accidentally perforated, possibly by the distal end of the 0.035′′ guide wire (Figure 2(a), online video 1 in Supplementary Material available online at http://dx.doi.org/10.1155/2015/725168). Visualization of the perforation was performed using DSA and the small side branch was wired by a 0.014′′ coronary guide wire (Figure 2(b), online videos 2 and 3). Repeated hemostasis by balloon occlusion of the popliteal artery (5.0*∗*40 mm angioplasty balloon, 3 times over 5 minutes, resp.) and by inflation of a blood pressure cuff proximally to the knee at 20 mmHg over the arterial pressure for another 3 times over 5 minutes, respectively, failed to stop bleeding out of the side branch. Thus, at that time two treatment options appeared reasonable, including (1) placement of a coated stent graft over the popliteal artery, covering the perforated side branch, or (2) placement of a coated small diameter coronary stent graft in the perforated side branch. In order to avoid long-term stent fracture of a coated stent graft placed in part of the mobile popliteal segment we decided to choose the second treatment option. Firstly, small diameter coronary balloon (Tazuna 1.5*∗*10 mm, Terumo Germany GmbH, Eschborn, Germany) was inserted over the wire in the perforated side branch and during balloon inflation with 14 bars bleeding ceased immediately (online video 4). Subsequently, implantation of a Direct-Stent stent graft (2.5*∗*19 mm, In Situ Technologies Inc., St. Paul, MN, USA) was performed in the perforated side branch, successfully and permanently stopping bleeding in this segment (Figures 2(c) and 2(d), online video 5). Our patient could be directly mobilized 4 hours after the intervention and did not report any local pain, paraesthesia, or intermittent claudication. Using colour doppler ultrasound the stent graft could be visualized one day after implantation, exhibiting normal blood flow. No signs of haematoma or other bleeding complications could be visualized by ultrasonography (Figures 2(e) and 2(f)).

## 3. Discussion

To our knowledge this is the first case reporting on the placement of coronary stent graft in a peripheral vessel for the management of a peripheral side branch perforation. Interventional angiologists or radiologists may consider such an option for complication management after injury of smaller vessels during peripheral percutaneous interventions. In the past years significant technical developments have occurred with endovascular therapy, which offer several distinct advantages over open surgical revascularization techniques in selected lesions [7]. Further specialization and novel options of complication management as described in our case may shift the treatment from surgical to even more endovascular treatment procedures in the future.

## Supplementary Material

Online Video 1: A small side branch of the popliteal artery was accidentally perforated.
Online Video 2 and 3: Visualization of the perforation was performed using DSA and the small side branch was wired by a 0.014“ coronary guide wire.
Online Video 4: During balloon inflation with 14 bars bleeding ceased immediately.
Online Video 5: Implantation of a Direct-Stent stent graft was performed in the perforated side branch, successfully and permanently stopping bleeding in this segment.
